# The Effect of Curcumin Supplementation on Anthropometric Measures among Overweight or Obese Adults

**DOI:** 10.3390/nu13020680

**Published:** 2021-02-20

**Authors:** Farah J. Alsharif, Yara A. Almuhtadi

**Affiliations:** Clinical Nutrition Program, Department of Community Health Sciences, King Saud University, Riyadh 11362, Saudi Arabia; falsharif@KSU.EDU.SA

**Keywords:** curcumin, turmeric, obesity, overweight, weight loss, body mass index, body weight, weight reduction

## Abstract

Over the past decades, the worldwide prevalence of obesity has dramatically increased, thus posing a serious public health threat. Obesity is associated with the development of comorbid conditions and psychological disorders. Several lifestyle interventions have been proposed to tackle obesity; however, long-term maintenance of these interventions often proves challenging. In addition, among the different types of diets there is still a debate about the optimal macronutrient composition that will achieve the best results in weight loss. Recently, several commonly used spices such as pepper, ginger, and curcumin have been shown to play a beneficial role in obesity management. Therefore, exploring the effects of certain herbs or dietary spices on obesity may be promising. Among these spices, curcumin, which is the primary component of the spice turmeric, has gained great interest for its multiple health benefits. Several randomized controlled trials have investigated the potential favorable effects of curcumin supplementation on anthropometric measures. The aim of this review is to evaluate the effect of curcumin supplementation on the anthropometric indices among overweight or obese adults.

## 1. Introduction

The prevalence of overweight and obesity has doubled globally since 1980, to an extent that almost one-third of the world’s population is now classified as overweight or obese [1,2]. Obesity poses a serious threat to public health, as it subsequently affects nearly all the physiological functions of the body [1,3]. It may adversely increase the risk for comorbid conditions such as type 2 diabetes mellitus (T2DM), cardiovascular diseases, and several types of cancers [1,3]. In addition to physical consequences, obesity presents negative psychological effects [4], all of which have an adverse impact on quality of life, work productivity, and health care costs [1].

Several lifestyle interventions have been proposed to tackle obesity, including reducing energy intake, increasing physical activity, and using behavioral therapy approaches [5,6]. These interventions have been shown to produce short-term reductions in body weight among overweight or obese individuals [7,8]. However, long-term weight loss and maintenance of these lifestyle modifications remain challenging [7,8]. In addition, there is still a debate about the optimal macronutrient composition that will achieve the best results in weight reduction among these different types of diets [9,10]. Recently, researchers have assessed the potential effects of commonly used spices in reducing body weight [11,12,13]. Some of these include red pepper, ginger, and curcumin which have been shown to play a beneficial role in the management of obesity [11,12]. Therefore, it might be rewarding to investigate the effects of dietary spices on obesity [5].

**Figure 1:**
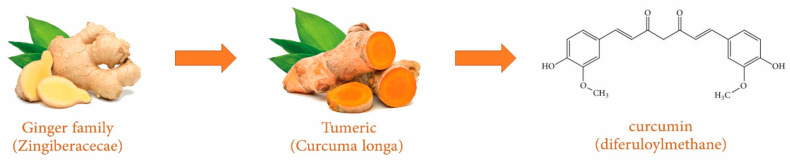
Chemical structure of curcumin.

Curcumin (diferuloylmethane) is a natural yellow crystalline polyphenol and the active ingredient of the spice turmeric (Curcuma longa), which belongs to the ginger family (Zingiberaceae) [14,15,16] (Figure 1). Curcumin has gained great interest for its antioxidant, anti-inflammatory, anticarcinogenic, antidepressant, antiarthritic [17], antidiabetic, hepatoprotective, and lipid-lowering properties [18].

Curcumin has been classified as pan-assay interference compounds (PAINS) [16]. These compounds exert their activity in several types of assays by interacting with the assay readout rather than through specific compound interactions. Curcumin manifests all PAINS-type behaviors such as aggregation, redox activity, metal chelation, membrane disruption, and fluorescence interference. This suggests that any report of curcumin activity in an assay that does not exclude or account for these behaviors could lead to false interpretations of curcumin activity [16].

Several systematic reviews of randomized controlled trials (RCTs) were conducted to evaluate the effects of curcumin supplementation on body composition including body weight, body mass index (BMI), and waist circumference [19,20,21,22,23]. Although most reviews included overweight or obese adults, some included participants with normal BMI [19,21,23]. In addition, some reviews were disease specific and only included participants with non-alcoholic fatty liver disease or metabolic syndrome [19,20,22]. Other recent reviews performed in overweight or obese patients showed conflicting outcomes [20,22]. This review aims to investigate and provide an update of the effect of curcumin supplementation on anthropometric measures including body weight, BMI, waist circumference, and percentage of body fat among overweight or obese adults.

## 2. Mechanism of Action

**Figure 2:**
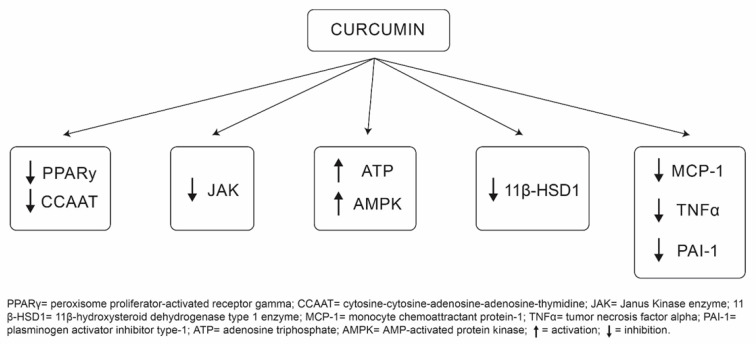
Curcumin mechanisms of action.

There are several potential mechanisms underlying the role of curcumin in obesity attenuation (Figure 2). First, curcumin can inhibit two important deoxyribonucleic acid (DNA) binding factors, also known as transcriptional factors, involved in adipogenesis [24,25,26]. These include expression of peroxisome proliferator-activated receptor gamma (PPARγ) and cytosine-cytosine-adenosine-adenosine-thymidine (CCAAT) [24,25,26]. Second, curcumin has been shown to inhibit the Janus Kinase enzyme (JAK), which plays a key role in the development of obesity [23,27,28]. In addition, because elevated levels of the stress hormone “cortisol” induce central obesity [20,29], curcumin downregulates the enzyme involved in its activation, known as 11β-hydroxysteroid dehydrogenase type 1 enzyme (11β-HSD1) [20,30]. Furthermore, curcumin can induce weight loss by suppressing pro-inflammatory cytokines such as monocyte chemoattractant protein-1 (MCP-1), tumor necrosis factor alpha (TNFα), and plasminogen activator inhibitor type-1 (PAI-1) [19,31]. Finally, curcumin can enhance energy expenditure and stimulate weight loss by increasing adenosine triphosphate (ATP) production and enhancing the activity of AMP-activated protein kinase (AMPK) [21,31,32,33]. In addition to curcumin’s role in tackling obesity, it has also been proposed that curcumin possesses epigenetic and antiproliferative properties. Curcumin has been found to decrease H3 histone glutathionylation, which helps in the inhibition of cellular proliferation. This occurs via the induction of G2M/cell-cycle arrest [34].

## 3. The Effect of Curcumin on Anthropometric Measures Results

Several RCTs have assessed the effect of curcumin supplementation on anthropometric measures among overweight or obese adults [17,26,35,36,37]. This review included only individuals aged over 18 years and had a BMI greater than or equal to 25 kg/m^2^ (Table 1). A major barrier to curcumin’s therapeutic efficacy is its low bioavailability and stability owing to its poor absorption, rapid metabolism, and excretion [5,38]. To overcome these limitations, several approaches have been proposed, which include using different doses and forms of curcumin [5,39,40,41]. Therefore, studies were categorized according to the amount and type of curcumin used: 1000 milligrams (mg) or less of curcumin per day, 1500 mg of curcumin per day, phytosomal form of curcumin, and other forms of curcumin.

### 3.1. Studies with 1000 mg or Less of Curcumin per Day

Four RCTs with a cross-over design and similar methodologies showed no significant differences between the curcumin supplementation and placebo groups in anthropometric parameters (Table 1) [15,36,42,43]. Among these studies, three included 30 obese adults aged 18–65 years with a BMI more than or equal to 30 kg/m^2^ [15,36,42]. Participants were randomized to receive either 1000 mg of curcumin per day with a bioavailability enhancer called “piperine” (*n* = 15) or placebo (*n* = 15) for one month. Following a two-week washout period, individuals were crossed over to rotate regimen for another month. In either of the groups, no significant differences were found between body weight and BMI, with *p* > 0.05 [15,36,42]. In addition, curcumin intake showed no influence on body fat percentage, waist, arm, and hip circumferences (*p* > 0.05) [15].

In the Niemen et al. study [43], participants were crossed over to alternate regimen using either turmeric or red pepper in 61 overweight or obese women. Participants were randomized to receive either 2800 mg of turmeric per day (equivalent to around 112 mg of curcumin) (*n* = 30) or placebo (*n* = 31) for one month. Following a two-week washout period, individuals were crossed over to receive either 1000 mg of red pepper per day (*n* = 31) or placebo (*n* = 30) for another month. The results revealed that neither the turmeric nor the red pepper group showed an influence on body weight and body fat percentage compared to the placebo group, with *p* > 0.05 [43]. Notably, all participants were females, which may restrict the generalizability of the results to the male participants.

Previous studies showed that an intake of 1000 mg or less of curcumin supplementation per day for one-month duration had no effect on the anthropometric measures even with the use of a bioavailability enhancer. This suggests that changes in the amount of curcumin, number of participants, and duration of the studies may produce different outcomes among overweight or obese adults.

### 3.2. Studies with 1500 mg of Curcumin per Day

Other researchers have looked at increasing the amount of curcumin to 1500 mg per day [41,44,45,46]. Saadati et al. [41] explained the reason behind the increment in the study dose was to overcome the low bioavailability and stability of curcumin. The study recruited 52 adults (50 used in the analysis) aged over 18 years with a mean BMI of 32.34 kg/m^2^. Participants were randomized to receive either 1500 mg of curcumin per day (*n* = 27) or placebo (*n* = 25) for three months. All participants were advised to perform physical activity and to follow an energy-balanced diet. Results showed significant reductions in mean body weight (−2.39 ± 3.45 vs. −3.9 ± 4.08) kg, BMI (−0.85 ± 1.3 vs. −1.33 ± 1.44) kg/m^2^, waist circumference (−5.259 ± 3.94 vs. −3.77 ± 6.19) cm, and hip circumference (−3.37 ± 6.08 vs. −3.39 ± 5.56) cm in both groups (*p* < 0.05). The mean waist-to-hip ratio reduced significantly only in the curcumin group compared to baseline (−0.02 ± 0.04; *p* < 0.05) [41]. Since all subjects were given lifestyle modification advices, this could probably explain the non-significant changes in anthropometric measures between the groups.

These findings are supported by the results of another study that used a similar methodology for ten-week duration [44]. In contrast to Saadati et al. [41], all participants were instructed not to change their dietary intake and physical activity during the study period. Results showed that, in comparison to the placebo group, the curcumin supplementation group had significant reductions in mean body weight (−0.64 ± 0.22 vs. 0.19 ± 0.37 kg; *p* = 0.04) and mean hip circumference (−1 ± 0.32 vs. 0 ± 0.46 cm; *p* = 0.01). Mean BMI decreased significantly in the curcumin group only as compared to baseline (−0.3 ± 0.03 kg/m^2^; *p* = 0.03). However, the result was not significant between the groups (+0.3 ± 0.03 vs. +0.1 ± 0; *p* = 0.08) [44]. Hence, excluding lifestyle interventions as a confounding factor could explain the improved anthropometric outcomes occurring in the curcumin group as compared to that in the placebo group.

Other researchers have investigated the effects of curcumin, with a dose of 1500 mg per day, on the anthropometric indices for a longer duration of six to nine months [45,46]. The first study included 213 adults with a mean age 59.37 years and a mean BMI of 26.96 kg/m^2^ [45]. All participants received lifestyle and dietary recommendations three months before randomization. Participants were then randomized to receive either 1500 mg of curcumin per day (*n* = 107) or placebo (*n* = 106) for six months. The results revealed a significant reduction in the percentage of visceral fat and total body fat in the curcumin group as compared to the placebo group (−3.01 vs. +0.11%; *p* < 0.05, −5.17 vs. +0.19%; *p* < 0.001, respectively). Although the study did not reveal any significant differences in waist circumference between the curcumin and placebo group (−2.5 vs. +0.2 cm; *p* > 0.05, respectively), it appeared to lower the waist circumference in the curcumin group only [45].

Similarly, the second study included 240 adults with a mean age of 57.44 years and a mean BMI of 26.64 kg/m^2^ for nine months [46]. The results showed that curcumin supplementation as compared to placebo caused significant reductions in body weight (−3.9 vs. +2.3) kg and waist circumference (−3.6 vs. +2.7) cm, with *p* < 0.05 [46].

Overall, it seems that a daily intake of 1500 mg of curcumin shows more promising results compared to a daily intake of 1000 mg or less in anthropometric measures among overweight or obese adults. Additionally, studies conducted with bigger sample size and for longer duration have produced better outcomes compared to previous studies [45,46]. Therefore, studies with larger sample sizes and for an extended duration are warranted to identify the effect of curcumin supplementation on the anthropometric measures among overweight or obese adults.

### 3.3. Studies with Phytosomal form of Curcumin

In addition to assessing the use of different doses of curcumin, other researchers have investigated its different forms. An effective way to enhance curcumin bioavailability is through its complexation with phospholipids in the form of phytosomes [18,40]. Five studies investigated the effect of phytosomal curcumin supplementation on anthropometric measures in overweight or obese adults [14,17,18,35,37].

The first study assessed the efficacy and tolerability of phytosomal curcumin in overweight individuals [35]. It included 44 Caucasian adults aged 18–70 years with a BMI in the range of 25–29.9 kg/m^2^. Prior to randomization, 127 participants received lifestyle interventions, including diet education sessions and advices for physical activity increase, for one month. Out of 127 participants, 44 achieved a weight loss of <2% and were included in the study. Participants were randomized to receive either 1600 mg of phytosomal curcumin with piperine (total containing 400 mg of curcumin) per day (*n* = 22) or placebo (*n* = 22) for one month. In addition, participants were encouraged to continue with the lifestyle interventions along with the treatment regimen. Anthropometric measures including body weight, BMI, body fat percentage, waist, and hip circumferences were recorded at baseline and at one and two months. Results showed that following the first month of lifestyle interventions, no significant changes have been shown on anthropometric measures. However, at the end of the second month, all measurements, except for the hip circumference were significantly reduced in the curcumin group as compared to that in the placebo group [35]. These findings may suggest the potential superiority of phytosomal curcumin supplementation accompanied by lifestyle interventions compared to lifestyle interventions alone.

Similarly, another study used the same form and amount of curcumin with piperine in 80 overweight adults for two months [18]. At baseline, all participants were advised to avoid unhealthy eating habits and to increase their physical activity. The results showed that the curcumin group had a significant reduction in BMI (−0.8) kg/m^2^ and waist circumference (−5) cm as compared to baseline (*p* < 0.05) [18].

Moreover, a study investigated the safety and efficacy of phytosomal curcumin supplementation in 102 adults (87 used for analysis), with mean age 46 years and a mean BMI of 29.02 kg/m^2^ [17]. Participants were randomized to receive either 1000 mg of the phytosomal form of curcumin (equivalent to 200 mg of curcumin) per day (*n* = 50) or placebo (*n* = 52) for two months. All participants received lifestyle and dietary advices before starting the study. The study showed that phytosomal curcumin was well tolerated and safe, with no reported severe adverse events. In addition, subjects in the curcumin group had a significant reduction in BMI (−0.99 ± 1.25) kg/m^2^ and waist circumference (−1.74 ± 2.58) cm as compared to those in the placebo group (−0.15 ± 1.31 kg/m^2^, −0.23 ± 3.49 cm; *p* < 0.05, respectively). This finding could be attributed to the role of curcumin in reducing physiological fatigue and enhancing physical performance [17].

Another study assessed the effects of phytosomal curcumin in 58 adults (45 used in the analysis) aged 25–65 years with a mean BMI of 29.05 kg/m^2^ [14]. Participants were randomized to receive either a capsule containing 250 mg of phytosomal curcumin (equivalent to 50 mg of curcumin) per day (*n* = 30) or a matched placebo capsule (*n* = 28) for two months. Study findings have shown no significant differences between the curcumin group and the placebo group in body weight (−1.2 vs. −1.56) kg, BMI (−0.43 vs. −0.36) kg/m^2^, and fat free mass (+0.11 vs. −1.08) kg (*p* > 0.05) [14]. The non-significant results may be due to the small dose of curcumin used in the study.

Mohammadi et al. [37] evaluated the effect of phytosomal and normal forms of curcumin on anthropometric measures. The study included 120 adults aged 18–65 years with a mean BMI of 30.85 kg/m^2^. Participants were randomized into three groups: 1. Receiving 1000 mg of phytosomal curcumin (equivalent to 200 mg of curcumin) per day (*n* = 40); 2. Receiving 1000 mg of normal curcumin per day (*n* = 40); and 3. A control group (*n* = 40) for six weeks. All participants were given isocaloric dietary advices throughout the trial. Results showed no significant differences in terms of body weight (−0.21, −1.31, −0.58) kg, BMI (−0.19, −0.30, −0.10) kg/m^2^, and waist circumference (−3.53, −3.31, −3.58) cm (*p* > 0.05) between the phytosomal, normal, and placebo groups, respectively [37]. Interestingly, curcumin supplementation in either the phytosomal or normal form did not reveal any significant effect on anthropometric measures.

In summary, studies with a daily phytosomal curcumin supplementation of 200–400 mg of curcumin given for a period of one to two months showed significant reductions on anthropometric indices among overweight or obese adults [17,18,35].

### 3.4. Studies with Other Forms of Curcumin

In addition to the use of phytosomal form of curcumin, other studies have investigated the application of different forms of curcumin for bioavailability enhancement purposes. These include nanocurcumin [5,11], a combination of curcumin with fenugreek fiber [47], and an amorphous, solid, form of curcumin [26].

The use of polymeric nanoparticles called nanocurcumin has been shown to increase curcumin bioavailability up to 22 times [48]. Polymeric nanoparticles are very small in size which increase their surface area for interaction with epithelial cells [49]. Various types of biodegradable polymers are used to prepare nanocurcumin which could be either natural or synthetic [50]. Two studies have assessed the use of nano-curcumin among overweight or obese adults [5,11]. The former aimed to investigate its effect on appetite scores and included 84 adults aged 25–50 years and had a BMI of 25–35 kg/m^2^ [11]. Participants were randomized to receive either 80 mg of nanocurcumin (*n* = 42) per day or placebo (*n* = 42) for three months. All participants received a reduced energy diet and were advised to increase their physical activity by a trained dietitian. Results revealed a significant reduction in mean appetite scores in both nano-curcumin and placebo groups (−1.33; *p* < 0.01 vs. −0.29; *p* < 0.03, respectively). In addition, there was a significant reduction in mean body weight (−2.8 vs. −2.4) kg and BMI (−0.9 vs. −0.8) kg/m^2^ in both groups (*p* < 0.01) [11]. The non-significant results between the two groups could be attributed to the lifestyle interventions that had been prescribed to participants.

Similarly, the latter study conducted in 2019 used the same methodology and the nano form of curcumin. It aimed to assess its effects on anthropometric indices, especially by investigating serum nesfatin levels [5]. Nesfatin is a neuropeptide produced in the hypothalamus and plays an important role in appetite regulation and fat storage [5]. Excess nesfatin administration in the brain leads to satiety, a decrease in body weight and body fat [51,52]. In contrast, lack of nesfatin leads to an increase in appetite, body weight and body fat [51,52]. Jazayeri-Tehrani et al. [5] study revealed that serum nesfatin significantly increased in the curcumin group (1.56 nanograms per milliliter (ng/mL); 95% confidence interval (CI): 1.52 to 1.59) as compared to that in the placebo group (0.18 ng/mL; CI: 014 to 0.21), with *p* < 0.05. Moreover, there was a significant reduction in waist circumference in the curcumin group (−5.8 cm; CI: −6.2 to −5.3) compared to that in the control group (−1.3 cm; CI: −1.7 to −0.8), with *p* < 0.05 [5]. The increased levels of serum nesfatin in the nano-curcumin supplementation group may be attributed to the improved waist circumference in the study by decreasing appetite and fat storage.

An enhanced formulation of curcumin combined with a soluble dietary fiber called fenugreek fiber has been shown to enhance curcumin’s bioavailability compared to that of its normal form [47,53]. Campbell et al. [47] study included 22 obese adult males that were randomized to receive either 1000 mg of formulated curcumin (equivalent to 316 mg of curcumin) with fenugreek fiber per day (*n* = 11) or placebo (*n* = 11) for three months. Participants were asked to maintain their lifestyle habits including their diet and physical activity levels throughout the trial. Study findings showed no significant differences between the formulated and placebo groups in BMI (+0.7 vs. +0.16) kg/m^2^ and body fat percentage (+1.3 vs. +0.51) % (*p* > 0.05). In addition, no significant differences were found between the groups in waist circumference (+0.39 vs. +0.14) cm and hip circumference (+0.89 vs. −0.05) cm (*p* > 0.05) [47].

It has been suggested that the crystalline structure of curcumin has a low aqueous solubility and tends to decrease its bioavailability [21,54]. Therefore, formation of the solid amorphous form of curcumin has been proposed to improve its absorption and water solubility [21,54]. A study investigated the effects of the amorphous form of curcumin on anthropometric parameters among 80 obese adults with a mean BMI of 31.09 kg/m^2^ [26]. Participants were randomized to receive either 500 mg of amorphous curcumin formulation (comprising 70 mg of curcumin) per day (*n* = 40) or a matched placebo (*n* = 40) for two months. Subjects in the curcumin group showed significant reductions in body weight (−1.81 ± 2.01) kg and BMI (−0.74 ± 0.85) kg/m^2^ compared to those in the control group (+0.49 ± 1.83 kg, + 0.02 ± 1.18 kg/m^2^; *p* < 0.05, respectively) [26]. Interestingly, the amorphous form of curcumin used in this study potentially helped in achieving a greater anthropometric effect by enhancing its absorption and bioavailability.

In conclusion, it seems that the use of nano and amorphous forms of curcumin has produced more promising results on anthropometric parameters compared to the combined form of curcumin with the fenugreek fiber among overweight or obese adults. However, more studies with different doses are needed to confirm their effects among overweight or obese adults.

## 4. Health Benefits

Curcumin has gained worldwide attention owing to its multiple health benefits. These benefits include its antioxidant, anti-inflammatory, anticarcinogenic, antidepressant, anti-arthritic [17], antidiabetic, hepatoprotective, and lipid-lowering actions [18]. Furthermore, curcumin has been shown to play a key role in the management of obesity by increasing basal metabolic rate and hence increasing energy expenditure [11,19,55]. Despite its health benefits, curcumin’s low bioavailability and stability, due to its poor absorption, rapid metabolism, and elimination, affect its therapeutic efficacy [5,38]. To optimize the health benefits, it is suggested to use a bioavailability enhancer such as piperine [39], phytosomal [40], nano [5], and amorphous curcumin [21,54].

## 5. Side Effects

In addition to the health benefits, curcumin has been also approved as “Generally Recognized as Safe” (GRAS) by the US Food and Drug Administration (FDA) [39,56]. According to the WHO research committee and the European Food Safety Authority (EFSA), as much as 3 mg curcumin per kg body weight can be taken daily [39,57]. Several trials have reported the safety of curcumin, with no adverse events [17,35,47]. However, some adverse side effects have been reported in other studies, including mainly nausea [5,26,37,45], abdominal discomfort [18,26,44] and constipation [37,46]. Other reported adverse events include itching, vertigo [46], hot flash [45], hypersensitivity [37], diuresis, increased duration, and volume of menstrual bleeding [15].

## 6. Limitations and Recommendations

Most of the studies in this review were conducted for a relatively short period, with an average follow-up duration of less than three months. None of the studies reached a follow-up duration of one year. In addition, small sample sizes were used in most studies, potentially leading to bias, and affecting the generalizability of the outcomes. Therefore, long-term curcumin supplementation with larger sample sizes is warranted to establish the real impact of curcumin on anthropometric measures. Furthermore, the instability and rapid degradation of curcumin—owing to its poor absorption, rapid metabolism, and excretion—may limit its therapeutic efficacy. To overcome these limitations, it would be useful to test higher doses with an enhanced form of curcumin such as phytosomal, nano, and amorphous curcumin. In addition, the potential false positive effects of curcumin due to its PAINS characteristics should be taken into consideration. Therefore, new approaches are warranted to accurately assess the health benefit impacts of curcumin supplementation. Moreover, to avoid any interference during the curcumin supplementation period, it would be crucial to exclude lifestyle interventions as a confounding factor.

## 7. Conclusions

In summary, the findings of the present review indicate that curcumin supplementation may exert beneficial effects against obesity among overweight or obese adults. These effects are mediated through its regulation of lipid metabolism by enhancing energy expenditure and suppressing transcriptional factors, enzymes and pro-inflammatory cytokines involved in adipogenesis. Except for a daily curcumin intake at 1000 mg or less per day, curcumin intake at 1500 mg or with different forms such as phytosomal, nano and amorphous curcumin showed positive improvements on anthropometric indices. However, further RCTs with larger sample sizes, longer follow-up durations, different doses, and forms of curcumin are needed to determine curcumin’s effect on anthropometric measures among overweight or obese adults.

## Figures and Tables

**Table 1:** Randomized controlled trials (RCTs) included in the review.

Author	Country	Sample Size (N) Dropouts (Males/Females)	Mean Age (years)	Mean BMI (kg/m^2^)	RCT (Design)	Intervention Duration (weeks)	Intervention Group/s (dose)	Control Group (dose)	Results (between the Groups)
Mohammadi et al., [15]; Sahebkar et al., [42]	Iran	*N* = 30 Dropouts = 0 5 M/25F	38.45	32.6	Double-blind, Cross-over	4	Curcumin (1000 mg/day) with Piperine (10 mg/day)	Piperine (10 mg/day)	Body weight (+0.2 vs. −1.4 kg, *p* = 0.23) (−0.7 vs. −0.3 kg, *p* = 0.23) BMI (0 vs. −0.6 kg/m^2^; *p* = 0.21) (−0.3 vs. −0.2 kg/m^2^; *p* = 0.21) Body fat percentage (−0.3 vs. −0.4%; *p* = 0.88) (−0.4 vs. −0.3%; *p* = 0.88) Waist circumference (−3 vs. −3.4 cm; *p* = 0.82) (−1.4 vs. −0.9 cm; *p* = 0.82) Arm circumference (−1.2 vs. −0.9 cm; *p* = 0.97) (−0.5 vs. −0.5 cm; *p* = 0.97) Hip circumference (−2.1 vs. −1.9 cm; *p* = 0.78) (−0.8 vs. −1.3 cm; *p* = 0.78)
Esmaily et al., [36]	Iran	*N* = 30 Dropouts = 0 6M/24F	38.32	33.3	Double-blind, Cross-over	4	Curcumin (1000 mg/day) with Piperine (10 mg/day)	Piperine (10 mg/day)	Body weight (*p* > 0.05) BMI (*p* > 0.05)
Nieman et al., [43]	USA	*N* = 64 Dropouts = 3 61F	56.7	34.6	Double-blind, Cross-over	4	Curcumin (112 mg/day)	White rice flour (N/A)	Body weight (+0.1 vs. +0.6 kg; *p* > 0.05) (+0.3 vs. +0.4 kg; *p* > 0.05) Body fat percentage (−0.5 vs. −0.4%; *p* > 0.05) (−0.7 vs. −0.8%; *p* > 0.05)
Saadati et al., [41]	Iran	*N* = 52 Dropouts = 2 27M/23F	45.66	32.34	Double-blind, Parallel	12	Curcumin (1500 mg/day)	Maltodextrin (N/A)	Body weight (−2.39 vs. −3.9 kg; *p* = 0.188) BMI (−0.85 vs. −1.33 kg/m^2^; *p* = 0.259) Waist circumference (−5.259 vs. −3.77 cm; *p* = 0.332) Hip circumference (−3.37 vs. −3.39 cm; *p* = 0.992) Waist to hip ratio (−0.02 vs. −0.008; *p* = 0.396)
Hodaei et al., [44]	Iran	*N* = 53 Dropouts = 9 22M/22F	59	28.7	Double-blind, Parallel	10	Curcumin (1500 mg/day)	Cooked rice flour (1332 mg/day)	Body weight (−0.64 vs. +0.19 kg; *p* = 0.04) BMI (+0.3 vs. +0.1 kg/m^2^; *p* = 0.08) Waist circumference (−1.2 vs. −0.43 cm; *p* = 0.26) Hip circumference (−1 vs. 0 cm; *p* = 0.01)
Chuengsamarn et al., [45]	Thailand	*N* = 213 Dropouts = 0 97M/116F	59.37	26.96	Double-blind, Parallel	24	Curcumin (1500 mg/day)	Starch (1500 mg/day)	Waist circumference (−2.5 vs. +0.2 cm; *p* > 0.05) Body fat percentage (−5.17 vs. +0.19%; *p* < 0.001) Visceral fat percentage (−3.01 vs. +0.11%; *p* < 0.05)
Chuengsamarn et al., [46]	Thailand	*N* = 240 Dropouts = 5 83M/152F	57.44	26.64	Double-blind, Parallel	36	Curcumin (1500 mg/day)	N/A	Body weight (−3.9 vs. +2.3 kg; *p* < 0.05) Waist circumference (−3.6 vs. +2.7 cm; *p* < 0.05)
Di Pierro et al., [35]	Italy	*N* = 44 Dropouts = 0 17M/27F	40.47	28.9	Parallel	4	Phytosomal curcumin (1600 mg/day) with Piperine (16 mg/day)	Phosphatidylserine (800 mg/day)	Body weight (*p* < 0.05) BMI (*p* < 0.01) Body fat percentage (*p* < 0.01) Waist circumference (*p* < 0.05) Hip circumference (*p* = 0.06)
Cicero et al., [18]	Italy	*N* = 80 Dropouts = 0 37M/43F	53.5	27	Double-blind, Parallel	8	Phytosomal curcumin (1600 mg/day) with Piperine (16 mg/day)	N/A	BMI (−0.8 vs. −0.5 kg/m^2^; *p* > 0.05) Waist circumference (−5 vs. −3 cm; *p* > 0.05)
Panahi et al., [17]	Iran	*N* = 102 Dropouts = 15 51M/36F	46.09	29.02	Parallel	8	Phytosomal curcumin (1000 mg/day)	N/A	BMI (−0.99 vs. −0.15 kg/m^2^; *p* = 0.003) Waist circumference (−1.74 vs. −0.23 cm; *p* = 0.024)
Chashmniam et al., [14]	Iran	*N* = 58 Dropouts = 13 27M/18F	42.15	29.05	Double-blind, Parallel	8	Phytosomal curcumin (250 mg/day)	N/A	Body weight (−1.2 vs. −1.56 kg; *p* > 0.05) BMI (−0.43 vs. −0.36 kg/m^2^; *p* > 0.05) Fat free mass (+0.11 vs. −1.08 kg; *p* > 0.05)
Mohammadi et al., [37]	Iran	*N* = 120 Dropouts = 11 86M/34F	38.72	30.85	Parallel	6	1. Phytosomal curcumin (1000 mg/day) 2. Normal curcumin (1000 mg/day)	Starch and lactose (N/A)	Body weight (−0.21, −1.31, −0.58 kg; *p* > 0.05) BMI (−0.19, −0.30, −0.10 kg/m^2^; *p* > 0.05) Waist circumference (−3.53. −3.31, −3.58 cm; *p* > 0.05)
Jazayeri-Tehrani et al., [5,11]	Iran	*N* = 84 Dropouts = 5 46M/38F	42.15	30.71	Double-blind, Parallel	12	Nano-curcumin (80 mg/day)	N/A	Body weight (−2.8 vs. −2.4 kg; *p* > 0.05) BMI (−0.9 vs. −0.8 kg/m^2^; *p* > 0.05) Body fat percentage (−1.3 vs. −1.2%; *p* > 0.05) Waist circumference (−5.8 vs. −1.3 cm; *p* < 0.05)
Campbell et al., [47]	USA	*N* = 22 Dropouts = 0 22M	26.27	33.23	Double-blind, Parallel	12	Formulated curcumin (1000 mg/day) with Fenugreek fiber (600 mg/day)	Fenugreek fiber (500 mg/day)	BMI (+0.7 vs. +0.16 kg/m^2^; *p* > 0.05) Body fat percentage (+1.3 vs. +0.51%; *p* > 0.05) Waist circumference (+0.39 vs. +0.14 cm; *p* > 0.05) Hip circumference (+0.89 vs. −0.05 cm; *p* > 0.05)
Rahmani et al., [26]	Iran	*N* = 80 Dropouts = 3 38M/42F	47.66	31.09	Double-blind, Parallel	8	Amorphous curcumin (500 mg/day)	N/A	Body weight (−1.81 vs. +0.49 kg; *p* < 0.001) BMI (−0.74 vs. +0.02 kg/m^2^; *p* = 0.002)
